# Role of Histone H3 Lysine 4 Methylation in Chromatin Biology

**DOI:** 10.3390/molecules30204075

**Published:** 2025-10-14

**Authors:** Bernhard Lüscher, Philip Bussmann, Janina Müller

**Affiliations:** Institute of Biochemistry and Molecular Biology, Medical Faculty, RWTH Aachen University, Pauwelsstrasse 30, 52074 Aachen, Germany; pbussmann@ukaachen.de (P.B.); janimueller@ukaachen.de (J.M.)

**Keywords:** acetylation, chromatin, COMPASS, gene expression, histone modification, methylation, PROTAC, RNAPII, transcription, transcription factor

## Abstract

Specific expression of genes is fundamental for defining the identity and the functional state of cells. Sequence-specific transcription factors interpret the information contained in DNA sequence motifs and recruit cofactors to modify chromatin and control RNA polymerases. This multi-step process typically involves several transcription factors and cofactors with different enzymatic activities. Post-translational modifications (PTMs) of histones are one key mechanism to control chromatin structure and polymerase activity and thus gene transcription. The methylation of histone H3 at lysine 4 (H3K4) is a modification of accessible chromatin, including enhancers and promoters, and also sites of recombination and some forms of DNA damage. H3K4 methylation is catalyzed by six lysine methyltransferase complexes, referred to as KMT2 or COMPASS-like complexes. These are important in processes related to transcription and contribute to recombination in T and B cells. PRDM9 and ASH1L are H3K4 methyltransferases involved in meiotic recombination and DNA repair, respectively. In transcription, H3K4 mono- and tri-methylation are located at enhancers and promoters, respectively. These modifications, either alone or in combination with other histone PTMs, provide binding sites for transcriptional cofactors. Through these sites, H3K4 methylation affects chromatin accessibility and histone PTMs, typically resulting in a favorable environment for transcription. H3K4 tri-methylation also recruits and regulates RNA polymerase II (RNAPII) complexes, which interact with KMT2 complexes, generating positive feedforward loops to promote transcription. Thus, H3K4 methylation has broad activities that are key to different chromatin-associated processes.

## 1. Introduction

Cell identity and physiology are specified by the pattern of expressed genes and the functionality of their products. This, together with additional mechanisms that control RNA processing, protein biosynthesis, and protein function allow us to adequately react to signals that, for example, impact proliferation and the cell cycle, differentiation, and specification, as well as to respond to different stressors, including DNA damage and viral infection. At the transcriptional level this encompasses the need for specific genes to be activated or repressed in response to signaling. Thus, determining how transcription is controlled is of long-standing, broad interest to understand physiological processes and alterations that occur in disease.

Gene transcription is fundamentally controlled by sequence-specific transcription factors (TFs) that recognize DNA sequences, referred to as response elements, typically near promoters and enhancers [1,2]. TFs recruit cofactor complexes that possess activities to modulate chromatin, including the positioning and the reorganization of nucleosomes [3,4]. This results in the transition between either more closed or more open chromatin (more generally referred to hetero- and euchromatin, respectively), which exist in multiple intermediates. These different states of chromatin compaction control the accessibility of enzymes to DNA that affect processes such as transcription. Compact chromatin reduces unscheduled transcription, such as from intragenic sites with TATA-like sequences, that is potentially toxic, for example, due to the generation of protein fragments [5,6]. Nucleosomes are the smallest units of chromatin with roughly 147 bp of DNA wrapped around an octamer of core histones [7]. Post-translational modifications (PTMs) of the core histones H2A, H2B, H3, H4, and their variants are an important aspect of nucleosome function as they can define functional states [3,8,9]. One of the challenges is to decipher the consequences of specific histone PTMs and even more so of combinations of PTMs for transcription but also for processes such as replication, DNA repair, and chromosome condensation and decondensation during mitosis. More than 450 PTMs have been described for histones. Typically, these are modifications owing to the addition of small chemical groups such as methyl, acetyl, or phosphate to various amino acid side chains [10]. Examples are the tri-methylation of histone H3 at lysine 9, which is associated with heterochromatin, and acetylation of multiple lysines of core histone typically found in euchromatin [11,12,13]. Together, these modifications offer a large combinatorial space potentially defining distinct functional states. Moreover, some amino acids can be modified by more than one chemical group, for example, multiple PTMs can occur on lysines, including methylation, acetylation, and ubiquitination [14]. This suggests that on the one hand, the positioning of enzymes that can write or erase specific PTMs and their ability to cooperate or compete to establish PTM patterns will affect the state of chromatin. On the other hand, readers that can interpret certain PTMs or combinations thereof are key to disseminating the information contained in the histone PTM pattern.

One of these PTMs, the methylation of lysine 4 of histone H3 (H3K4), has attracted broad attention because of its link to various processes, including gene transcription, DNA repair and recombination [4,15,16,17,18,19]. Methylation of lysine was discovered more than 60 years ago and studies on histones helped establish this as a PTM [20]. Despite the difficulties in defining functional relevance for many years, it led to the farsighted hypothesis that lysine methylation “… may affect the capacity of the histones to inhibit ribonucleic acid synthesis in vivo” [21]. Another key observation was the finding that H3K4 methylation correlates with transcription [22]. This, in combination with other studies, resulted in an influential review that formulated the concept of histones as protein interaction platforms controlled by PTMs [23], a basic concept that has been broadly validated. The combinatorial use of different histone PTMs is often referred to as the histone code [23,24,25].

In this review we focus primarily on the role of H3K4 methylation in transcription. We address questions including its functional relevance for chromatin remodeling, histone modification, and gene transcription. We discuss the intimate interplay with the RNA polymerase II complex and the resulting consequences on transcription. Moreover, we summarize the current knowledge on how specificity is achieved regarding both the positioning and the reading of H3K4 methylation for disseminating the information associated with this mark. We further discuss combinatorial effects with other histone marks. In the context of the large number of PTMs associated with core histones, it is not surprising that H3K4me3 has multiple functions dependent on its positioning and combination with other core histone PTMs. Together, the available data suggests that H3K4 methylation is a key modification controlling functions in transcription in combination with many other histone marks.

## 2. Writers and Erasers of H3K4 Methylation

### 2.1. H3K4 Methyltransferases

**Figure 1:**
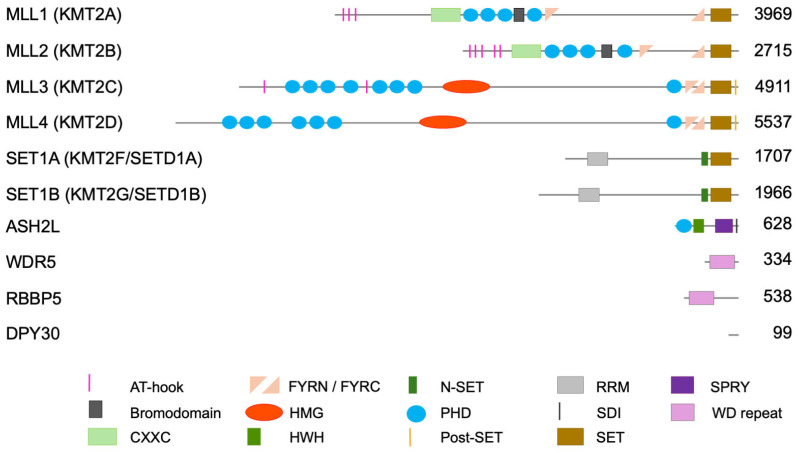
Domain organization of human subunits of KMT2 complexes. Shown are the six catalytic subunits (MLL5/KMT2E is not shown as thus far no catalytic activity has been reported for this KMT2 member), and the four core subunits that form the WRAD complex, which is associated with and necessary for the catalytic activity of all six KMT2 subunits. AT-hooks, adenosine-thymidine-hook; CXXC, Zinc finger-CXXC domain; FYRN/C, phenylalanine and tyrosine rich region (N- and C-terminal); HMG, high mobility group; HWH, helix-wing-helix domain; N-SET, N-terminal of SET; PHD, plant homeodomain; Post-SET, C-terminal of SET; RRM RNA recognition motive; SDI, Sdc1-Dpy-30 interaction; SET, Su(var)3-9, Enhancer-of-zeste and Trithorax; SPRY, spla and the ryanodine receptor domain; WD repeat, tryptophan-aspartic acid repeat. The numbers on the right indicate amino acids of human proteins. The information regarding domains is retrieved from https://www.ncbi.nlm.nih.gov/protein/ (accessed on 18 August 2025).

**Figure 2:**
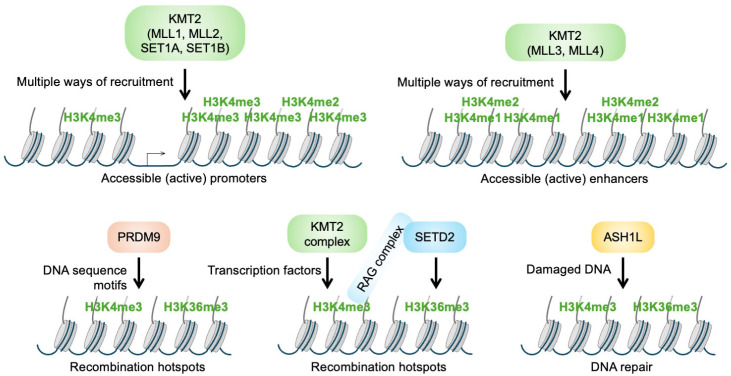
Localization and specificity of different enzymes that can methylate histone H3K4. (**Upper panels**) KMT2 complexes containing MLL1, MLL2, SET1A, or SET1B locate primarily to promoter regions and tri-methylate histone H3K4. The complexes that contain MLL3 or MLL4 locate to enhancers and primarily mono-methylate histone H3K4. ‘Multiple ways of recruitment’ refers to the different possibilities that allow the positioning of KMT2 complexes in distinct chromatin regions. Recruitment includes binding to sequence-specific transcription factors, to RNA polymerase II, to CpG islands, and to histone modifications (for more details, see the text). Additional mechanisms to recruit KMT2 complexes include direct binding to DNA and through long noncoding RNAs. (**Lower panels**) H3K4me3 is also associated with recombination and DNA repair. For these functions, a combination with H3K36me3 is necessary. PRDM9, which can modify both sites, binds to DNA sequences associated with recombination hotspots (**left**). For V(D)J recombination in T and B cells, KMT2 complexes are probably recruited by TFs. The resulting H3K4me3 is read by the RAG complex that then tri-methylates H3K36 by recruiting SETD2 (**middle**). Some forms of DNA damage provoke recruitment of ASH1L, which can tri-methylate both H3K4 and H3K36 (**right**).

The observation that H3K4 methyltransferase activity is detected in transcriptionally active macronuclei of Tetrahymena, but not in transcriptionally inert micronuclei [22], was important to develop the hypothesis that this PTM is associated with transcription. This led to the identification of yeast Set1, a class 2 lysine methyltransferase (KMT2), with a catalytically active SET domain that transfers methyl groups from SAM onto lysine side chains [26,27,28]. In yeast, Set1 appears to be the only KMT2 enzyme, which is part of a complex referred to as COMPASS [29]. In mammals, H3K4 methylation is catalyzed by complexes that contain one of six KMT2 catalytic subunits, MLL1/KMT2A, MLL2/KMT2B, MLL3/KMT2C, MLL4/KMT2D, SET1A/KMT2F, and SET1B/KMT2G, large multidomain proteins (Figure 1) [28]. All KMT2 or COMPASS-like complexes possess an essential common core complex of the four conserved subunits WDR5, RBBP5, ASH2L, and DPY30, referred to as the WRAD complex, while additional subunits are specific for individual complexes (Table 1). The WRAD complex is necessary for efficient methyltransferase activity. MLL3 and MLL4 complexes catalyze mono-methylation and possibly di-methylation, while the other four complexes di- and tri-methylate H3K4 (Figure 2). In the context of transcription, H3K4me1 and H3K4me3 are primarily found at enhancers and at promoters, respectively, while the role of H3K4me2 is less well described but generally overlaps with active chromatin [4,15,17,30]. Of note, recent findings suggest that H3K4me2 may be relevant for the function of some enhancers, but repressive functions have also been noted [31,32,33]. Also, H3K4me2 might be a transient intermediate between mono- and trimethylation. Because of the sparse available data on the functional role of H3K4me2, the focus of the discussion here will be on H3K4me1 and H3K4me3.

The above described KMT2 family enzymes are primarily associated with gene transcription and with H3K4 methylation at enhancers and promoters. In addition, H3K4me3 is also linked to sites of recombination and DNA repair, which are catalyzed by distinct enzymes (Figure 2) [18,19]. In meiotic recombination, PRDM9 tri-methylates H3K4 [19,34,35]. PRDM9, which is expressed in germ cells and some tumors, recognizes specific sequence motifs that define recombination hotspots. In addition to H3K4me3, PRDM9 modifies other lysines, including H3K36, resulting in the duale H3K4me3/H3K36me3 modification of recombination sites (Figure 2). H3K4me3 is also important for V(D)J recombination in B and T cells, a key process in adaptive immunity [36], again in combination with H3K36me3. In these cells, H3K4me3 is linked to promoters and probably KMT2-dependent, while H3K36me3 is catalyzed by SETD2. Additional determinants are recombination signal sequences recognized by the RAG complex, besides its ability to read H3K4me3 and to recruit SETD2 [37,38]. Thus, as for meiotic recombination, a combination of H3K4me3 and H3K36me3 appears to promote V(D)J recombination. It is of note that during recombination, DNA double-strand breaks occur, suggesting that H3K4me3/H3K36me3 might also be linked to DNA repair. Indeed, ASH1L, which can modify both sites, recognizes certain forms of DNA damage [18,39].

### 2.2. H3K4 Demethylases

The identification of lysine demethylases was challenging, which led to the suggestion that either histone tails might be clipped to remove methylated histone tails or methylated histones might be replaced entirely (discussed in [40]). Since the identification of LSD1 as a H3K4me2 demethylase, demonstrating that lysine methylation can be dynamic [40], many additional demethylases have been identified [41]. This includes several H3K4 demethylases, which belong to class 1, 2, and 5 lysine demethylases (KDMs). While class 1 and 2 enzymes, i.e., KDM1A, KDM1B, and KDM2B (LSD1 and 2 and FBXL10, respectively) preferentially hydrolyze mono- and di-methylated H3K4, KDM5A-D (JARID1A-D) demethylate H3K4me2 and me3 [41,42]. The latter is well documented by either depleting or inhibiting KDM5 enzymes, which strongly stabilizes H3K4me3 when KMT2 complexes are inactivated [43,44]. Of note, KDM1 enzymes belong to the FAD-dependent amine oxidases, while KDM2 and KDM5 enzymes contain a Jumonji C domain and are Fe(II) and 2-oxoglutarate-dependent dioxygenases [41], some of which are targeted by small molecule inhibitors potentially relevant for clinical applications [45].

In summary, multiple writers and erasers of H3K4 methylation have been identified. While KMT2 family members are associated with transcription and chromatin organization, as further detailed below, PRDM9 and ASH1L are linked to recombination and DNA repair. They define, together with the erasers, H3K4 methylation as a dynamic PTM. The findings also suggest that H3K4 methylation has different roles depending on the genomic location and the local chromatin context. Thus, key questions are how local H3K4 methylation is achieved and what the consequences are of the different H3K4 methylation states.

## 3. Locating KMT2 Complexes to Distinct Chromatin Sites

**Figure 3:**
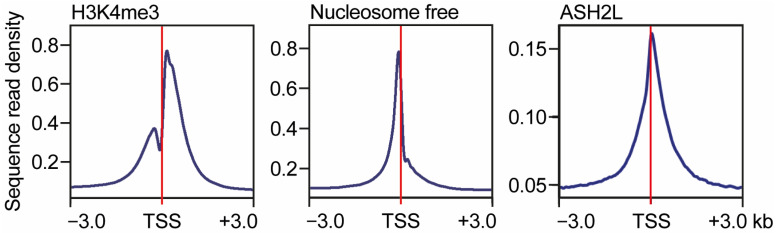
Pattern of histone H3 lysine 4 methylation at promoters. Summary of ChIP-seq experiments performed with antibodies selective for H3K4me3 from cross-linked chromatin obtained from murine embryo fibroblasts (**left panel**). Parallel ATAC-seq experiments were used to determine nucleosome-free or -depleted regions (**middle panel**). ChIP-seq experiments were carried out with antibodies selective for the ASH2L core subunit of KMT2 complexes (**right panel**). The region around transcriptional start sites is shown (TSS ± 3 kb). The data is taken from [51].

Genome-wide analyses of H3K4 methylation demonstrate that H3K4me1 is linked with enhancer regions and H3K4me3 with active promoters [17,46]. While at enhancers the H3K4me1 pattern is typically broad, the H3K4me3 pattern at promoters is often biphasic around the TSS (Figure 3) [44,47,48,49,50,51,52]. Moreover, the region downstream of the TSS carrying H3K4me3 can be either rather narrow or extended, the latter is referred to as broad H3K4me3 domains, which are linked to cell type-specific gene transcription [53]. One possible reason for the biphasic pattern of H3K4me3, which is also seen for H3K27ac, another modification associated with active promoters [51,54], is the nucleosome-depleted regions (NDRs) that have been noted at and upstream of the TSS of transcribed genes (Figure 3). Establishing NDRs is thought to be important to provide sufficient space for the positioning of the DNA-dependent RNA polymerase II (RNAPII) pre-initiation complex (PIC) [55,56]. Indeed, H3K4me3 at promoters correlates with reduced nucleosomal occupancy and enhanced acetylation of, for example, H3K27 and H3K9 [51,55,56,57,58]. Thus, NDRs with reduced nucleosomal occupancy may suffice to result in the reduction in H3K4me3 signals precisely in these promoter proximal regions.

### 3.1. Sequence-Specific Transcription Factors Interact with KMT2 Complexes

**Scheme 1:**
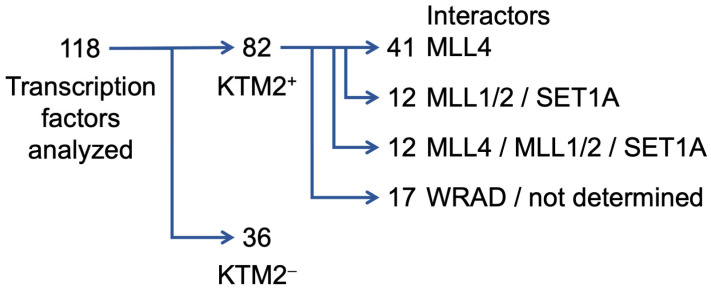
Multiple interactions between sequence-specific transcription factors and subunits of KMT2 complexes have been identified. These are summarized in Supplementary Table S1. Of the 118 TFs that are listed, 82 interact with one or more subunits of KMT2 complexes (indicated as KMT2^+^), while for 36, no interaction has been documented (indicated as KMT2^−^). Of the 82 TFs that show interaction with KMT2 complex subunits, 41 interact with the mono-methylating MLL4 and thus are potentially forming complexes at enhancers, while 12 TFs bind catalytic subunits that tri-methylate H3K4 and may be linked to promoter proximal response elements. Interestingly, 12 TFs can apparently interact with both mono- and tri-methylating catalytic subunits. Finally, 17 TFs bind either to WRAD components or the specific subunit(s) that mediate the interactions have not been documented. Of note, for most of these interactions it remains unclear whether these occur on chromatin, i.e., whether TFs recruit KMT2 complexes to their respective response elements, and whether these interactions have functional consequences (see also the text).

**Figure 4:**
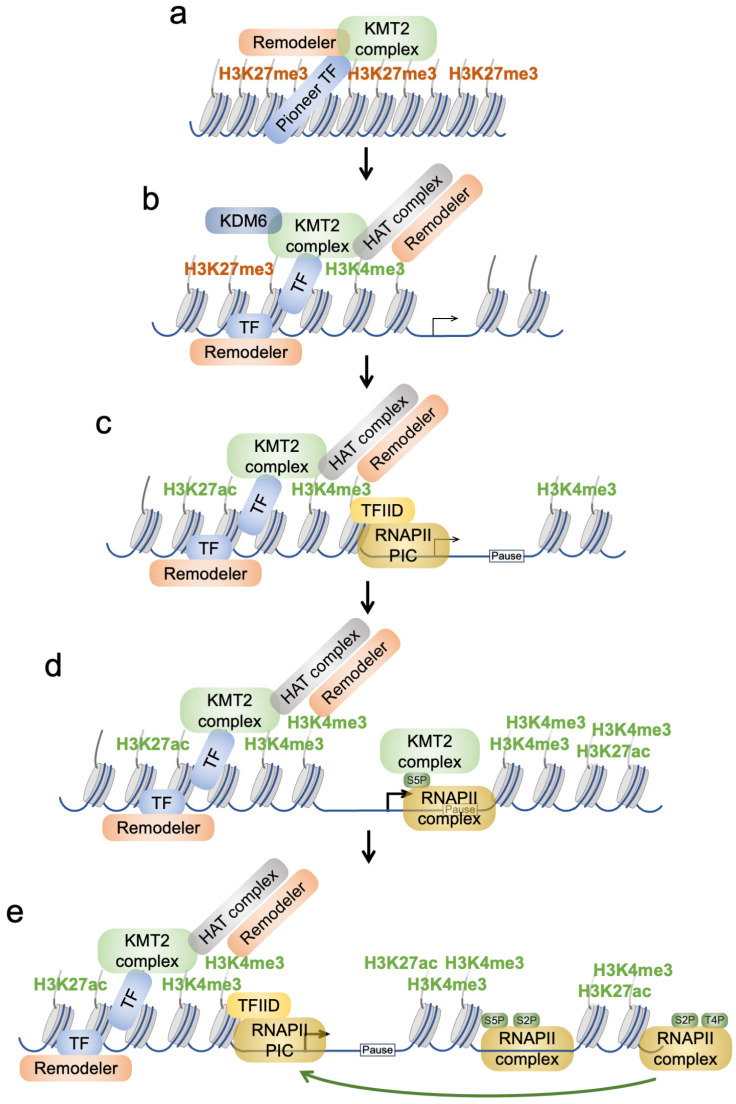
Model of the role of H3K4 methylation in promoting gene transcription. (**a**) A silenced region of chromatin with H3K27me3 is shown. Pioneering TFs are capable of binding to little accessible chromatin and promote chromatin opening. This is achieved by the recruitment of cofactors, such as chromatin remodeling and KMT2 complexes. (**b**) A consequence is chromatin opening, which allows for the binding of additional TFs. KMT2 complexes can interact with KDM6 enzymes that can demethylate H3K27me3 and thus make acetylation possible via histone acetyltransferases (HATs). These can be recruited by both sequence-specific TFs and KMT2 complexes. (**c**) This results in demethylation of H3K27, tri-methylation of H3K4, and acetylation of H3K27, as well as other lysines (not shown). Together with chromatin remodelers, the chromatin at core promoters is becoming accessible. This is thought to result in a nucleosome-depleted region that provides sufficient space for RNAPII pre-initiation complex recruitment and binding. This is favored by the ability of the TFIID complex, a subcomplex of the PIC, to read H3K4me3 through its TAF3 subunit. (**d**) Upon phosphorylation of serine 5 (S5P) of the C-terminal domain of RNAPII, the polymerase complex can translocate to the transcriptional pause site. S5P may be affected by KMT2 complexes. Moreover, this promotes binding of KMT2 complexes to RNAPII. This localization is further aided by the ability of KMT2 complexes to bind to unmethylated CpG dinucleotides, which are particularly abundant in CpG island promoters (the majority of human and murine promoters possess CGIs). Overall, this leads to further H3K4me3 and H3K27ac as well as other modifications. (**e**) Further progression from the pause site of the RNAPII complex is dependent on S2P. Termination of transcription is characterized by S2P and T4P of the CTD. The high levels of H3K4me3 in the promoter proximal regions is thought to promote reinitiation of gene transcription, which requires changes in the RNAPII modification pattern, a process that is only partially understood. Together, this model suggests that KMT2 complexes and H3K4me3 play key roles in chromatin remodeling and opening, recruitment of writers and erasers, RNAPII complex recruitment, and reinitiation of transcription. Not all the histone marks, proteins, and interactions are shown, which are discussed in the text, for clarity. The focus here is on promoters. We imagine that the opening of chromatin at enhancers may also be driven by pioneering TFs and the recruitment of chromatin remodelers and KMT2 complexes.

How are these patterns of H3K4 methylation established, in other words, how are the relevant writers and erasers positioned in chromatin? Various forms of targeting KMT2 complexes to specific regions in the genome have been described, including sequence-specific TFs, chromatin modifications, DNA sequences, and RNAPII, as previously summarized [30,59,60]. First, we will focus on sequence-specific TFs. These hold key roles to initiate and control gene transcription by binding to DNA response elements with defined sequence information in enhancer and promoter regions. Therefore, it seems likely that TFs are important to recruit H3K4 methyltransferases to regulate transcription. Indeed, a number of TFs, including some with pioneering activity, such as KLF4, OCT4, and GATA3 [61,62], have been shown to interact with KMT2 complexes (Figure 4). In one study, applying proximity-dependent biotinylation (BioID), in which 109 TF-biotin ligase fusion proteins were expressed and examined, 65 TFs resulted in the modification of at least one KMT2 complex subunit [63]. Additional interactions with TFs have been described (Supplementary Table S1, summarized in Scheme 1) [30,64]. Of note, BioID labeling is not evidence for direct interaction and labeling depends on the geometry of complexes formed. In general, proximity is sufficient, as the name of the method suggests. Thus, the interaction of a TF-biotin ligase fusion protein with a complex may be indirect but may suffice to label complex subunits. Alternatively, the interaction of a TF-biotin ligase fusion protein may be specific for one subunit but can result in biotinylation of this, another, or even multiple subunits. Moreover, lack of biotinylation does not exclude interaction, as the geometry of the interactions may not position the ligase sufficiently close to an acceptor lysine side chain. Nevertheless, these findings suggest that KMT2 complexes are potentially recruited to enhancers and promoters by at least some sequence-specific TFs (Figure 4). Because TFs can act early in gene transcription, particularly pioneering TFs, they might provide a first wave of H3K4 methylation. MLL4 has been identified more frequently compared with other catalytic subunits, an indication that many TFs-KMT2 complexes are linked to enhancers and H3K4me1 (Supplementary Table S1 and Scheme 1).

Despite these numerous interactions, the findings do not provide information on whether KMT2 complexes are recruited to specific chromatin sites by TFs. Thus, one important question to address is whether the interactions of TFs with KMT2 complexes occur on chromatin. Such information would provide evidence that TFs can contribute to the local enrichment of KMT2 complexes, although this does not mean that this is biologically relevant. So far, information about colocalization has been demonstrated in only a few cases. One example is c-MYC, which not only coimmunoprecipitates H3K4 methyltransferase activity, but colocalizes with ASH2L on chromatin [65]. In this study, binding of c-MYC was suggested to occur at response elements of target genes that contain c-MYC/MAX binding sites [66]. Moreover, c-MYC interacts with several subunits of KMT2 complexes both in vitro and in cells [63,65,67]. Thus, c-MYC is an example of a sequence-specific TF that can recruit KMT2 complexes to its response elements.

While for many years direct interactions have been considered as a key mechanism of sequence-specific TFs to recruit cofactors to chromatin, recent observations provide evidence that TFs interact through intrinsically disordered regions (IDRs), frequently functioning as transactivation domains. Such interactions can result in the formation of condensates through liquid–liquid phase separation, which not only help to position TFs potentially independent of DNA binding sites, but also to provide an environment to recruit cofactors [68,69,70,71]. Thus, it is likely that KMT2 complexes are also recruited by interaction through IDRs and their integration into condensates. It is difficult to estimate how frequent such interactions are, but most likely, additional TFs will be identified to recruit KMT2 complexes beyond the above summarized interactions. As a note of caution, such interactions based on dynamic condensates, particularly when specific sites in chromatin are evaluated, may be difficult to detect with methods such as ChIP experiments.

### 3.2. Positioning of KMT2 Complexes near Promoters

Many response elements are positioned in promoter proximal regions, in addition to enhancers that can be far from TSSs, often within a few hundred base pairs upstream of the TSS [72,73]. One might expect that H3K4 methylation signals are detected in such promoter proximal upstream regions. However, the biphasic H3K4me3 pattern observed at promoters, as discussed above, seems not to be compatible with this prediction. ChIP signals typically do not extend to upstream promoter regions. Among the reasons why such signals might not be detected is that the interactions of sequence-specific TFs with their response elements are generally described as short-lived, and the recruitment of cofactors such as KMT2 complexes may only be transient [74]. This makes it difficult to detect specific ChIP signals upstream of the TSS. Nevertheless, transient recruitment of KMT2 complexes by TFs to promoter proximal response elements might be functionally sufficient for an initial H3K4me3 signal (Figure 4).

While the broad H3K4me1 signals at enhancers correlate well with the multiple binding sites for sequence-specific TFs typically found at such DNA elements, the strong and biphasic H3K4me3 pattern at promoters may depend on the localization of KMT2 complexes by other means. In addition to recruitment through sequence-specific TFs, multiple additional mechanisms have been described, including interaction with the RNAPII complex and with non-methylated CpG dinucleotides that are abundant in CpG islands (CGIs) [30]. Before discussing these interactions, it is worth addressing the positioning of the KMT2 complexes at promoters. Unlike H3K4me3, all four WRAD components and the catalytic subunits are located close to the TSS, but do not show a biphasic pattern (Figure 3) [43,44,50,51,52,75,76,77,78,79,80]. This resembles the positioning of RNAPII at promoters near the TSS and the pause site, suggesting a close interaction of KMT2 and RNAPII complexes.

Indeed, interaction with the RNAPII can occur through WDR82, a subunit that is found in SET1A/B complexes, possibly dependent on the serine 5 phosphorylated (S5P) C-terminal domain (CTD) of RNAPII [81,82,83,84]. This represents the activated but not yet elongating enzyme, which is positioned close to the TSS and the pause site [85], and thus is consistent with the localization of KMT2 complex subunits as discussed above (Figure 4). Moreover, this is also consistent with the NDR at active promoters, which allows for efficient PIC binding [56]. Unlike the above-discussed difficulties that may be relevant in detecting KMT2 complexes and H3K4 methylation at response elements, the RNAPII is well-positioned close to the TSS of transcribed genes at the pause sites. Moreover, the CTD of RNAPII possesses multiple S5P binding sites. Together, this can provide multiple binding sites for KMT2 complexes at core promoters and thus explain the strong signals obtained for KMT2 complex subunits and for H3K4me3 in ChIP experiments.

### 3.3. Positioning of KMT2 Complexes by Binding to CpG Island Promoters

In addition to binding to the RNAPII CTD, SET1A/B complexes possess a CFP1 (CXXC zinc finger protein 1) subunit, which interacts with non-methylated CpGs [86,87,88]. Lack of CFP1 strongly reduces H3K4me3, particularly at promoters with CGIs [89,90]. CFP1 also contains a PHD domain that reads H3K4me3 [91,92,93]. Moreover, MLL1/2 possess CXXC domains that can interact with non-methylated CpGs and, thus, also promote localization to CGIs [94]. The presence of the CXXC domains in MLL1/2 and the association of SET1A/B with CFP1 is thought to favor association with promoters, while MLL3/4, which lack such domains, are preferentially bound to enhancers [95]. The importance of the interplay of CGI promoters with H3K4me3 is further supported by the observation that this histone mark prevents binding of DNA methyltransferases and thus precludes CpG methylation [9]. Together, these studies support an intimate interaction of KMT2 complexes with CGI promoters and RNAPII [96]. This may promote H3K4me3 but also maintain and spread this mark as part of feedback mechanisms, further emphasized in the discussion below.

## 4. Readers of H3K4 Methylation and Functional Consequences

An important question is what the consequences are of the H3K4 methylation mark, which is associated with gene transcription. Methylation does not neutralize the positive charge of the lysine side chain at physiological pH, unlike, for example, acetylation. Nevertheless, the methyl groups will affect its electrostatic interactions due to altered hydrophobic and steric properties. Reader domains such as chromodomains, Tudor domains, and PHD domains possess a hydrophobic cage that, together with additional features such as cation-π stacking, recognizes methylated lysines. Additional specificity may be obtained by recognizing other characteristics such as the underlying amino acid sequence or a combination of histone modifications [97]. In the following, we discuss selected readers, their relevant domains that recognize different H3K4 methylation states, and functional consequences. For a more comprehensive list of reader domains, we refer to published reviews [17,98,99].

### 4.1. Recruiting Cofactors by H3K4me3

The positioning of H3K4 methylation suggests that it affects the functionality of both enhancers and promoters. This can be achieved by providing binding sites for cofactors. Indeed, H3K4me3 is read by proteins associated with chromatin remodeling as well as controlling other histone marks. For example, the nucleosome remodeling factor NURF can interact with H3K4me3 via a PHD domain of its largest subunit BPTF [100,101,102,103,104]. CHD1, an ATP-dependent chromatin remodeler, uses chromodomains to recognize H3K4me3 [103,105,106]. CHD1 exhibits a preference for binding to GC-rich active promoters, to preserve hypertranscription and safeguard against double-strand breaks [107]. Thus, H3K4me3 may participate in reorganizing and opening chromatin at promoters to facilitate access of the RNAPII complex, again consistent with the decreased accessibility of promoters upon loss of ASH2L [51,58].

In addition, ING proteins contain H3K4me3-reading PHD domains and are part of histone acetyltransferase complexes, potentially enhancing gene expression [108,109,110]. The SGF29 subunit of the multifunctional SAGA complex, which can acetylate multiple lysine residues of core histones, interacts with H3K4me3. SAGA also interacts with TFIID, which in turn binds to H3K4me3, as discussed below [111,112,113,114,115]. This suggests that the binding of SAGA to H3K4me3 contributes to both promoter accessibility and PIC loading, thereby promoting transcription. PHF2 is a H3K9 demethylase; H3K9me3 is a modification typically associated with heterochromatin [11,116]. PHF2 binds to H3K4me3 through its PHD domain and thus may also promote an environment favorable for transcription [117]. In summary, these findings provide evidence for a broad role of H3K4me3 in controlling gene transcription by providing binding sites for transcriptional cofactors that positively affect chromatin remodeling and promote additional histone marks to further transcription (Figure 4).

### 4.2. Interactors at Enhancers and Response Elements

Not only H3K4me3 at promoters but also H3K4me1 at enhancers have been linked to remodelers. H3K4me1 promotes the association of SWI/SNF chromatin remodeling complexes [118]. It is also of note that some of the above-mentioned sequence-specific TFs, for example, KLF4, OCT4, c-MYC, and GATA3, which can interact with KMT2 complexes, also bind SWI/SNF [61,62,119,120,121,122]. Finally, SWI/SNF and KMT2 complexes interact directly [123], suggesting that at enhancers, but possibly also at promoters, different SWI/SNF complexes cooperate with H3K4 methylation [62]. Together, these findings provide ample evidence of a close interplay of H3K4 methylation and other cofactor activities.

### 4.3. Recruitment and Regulation of the RNAPII Complex

The strong association of H3K4me3 with promoters suggests an intimate relation with the RNAPII complex. The TFIID complex, composed of the TATA-box binding protein TBP and associated factors (TAFs), is important for assembling the PIC (Figure 4) [124]. One of the TFIID subunits, TAF3, reads H3K4me3 through its PHD domain, indicating that H3K4me3 is directly contributing to RNAPII recruitment [111,112,125,126,127]. However, a recent study challenged these findings. PROTAC-induced loss of RBBP5 did not affect TFIID binding, while another H3K4me3 reader, BAP18, was substantially reduced [43]. Despite the lack of consequences for TAF3 loading, RNAPII at promoters was reduced, indicating that some downstream event is affected upon markedly reduced H3K4me3 [43]. It is argued that it is not PIC formation that is affected but progression to the pause site. In another study, the residence time at pause sites downstream of the TSS was increased and further elongation decreased upon inactivation of KMT2 complexes [44]. Together, these observations suggest that assembly and/or activation of RNAPII complexes may be less efficient. Although H3K4me3 was strongly reduced, residual H3K4me3 may remain, leaving the question open at which level of promoter-associated H3K4me3 the recruitment and/or the activation the RNAPII complex is affected. Indeed, decreased RNAPII recruitment is only seen when H3K4me3 is strongly reduced [43]. Moreover, upon loss of RBBP5, RNAPII-S5P was decreased [43], possibly indicating that the TFIIH subcomplex with the CTD-S5 kinase cyclin H/CDK7 is less active or not sufficiently recruited [128]. The reduced chromatin accessibility near promoters upon loss of ASH2L may affect proper loading of the RNAPII complex [51,58]. Moreover, low S5P may explain the reduced progression to the pause site, but also the decrease in transcription may be affected because S5P serves to recruit the RNA capping machinery and thus affects mRNA stability [124,128,129].

Together, the intimate interaction of H3K4me3 and the RNAPII complex is striking. Not only does H3K4me3 participate in RNAPII complex loading, but RNAPII also recruits KMT2 complexes. This argues for a feedforward loop to enhance both H3K4 methylation and RNAPII binding to core promoters. This may be further accentuated at CGI promoters. One possibility is that these interactions facilitate the re-initiation of RNAPII, thereby sustaining high levels of gene transcription [130]. This is also compatible with H3K4me3 being both instructive as well as a consequence of transcriptional initiation [131]. At low levels, for example, due to TF-dependent loading of KMT2 complexes, H3K4me3 might be instructive for the initial RNAPII complex recruitment. This would then provoke high levels of H3K4me3, which in turn would instruct and support the reinitiation of transcription.

### 4.4. Interplay of H3K4 Methylation with Other Histone Marks

One of the key questions is how specificity at various levels is achieved by controlling chromatin and transcription. Sequence-specific TFs are clearly important to mediate the deposition of histone marks, including H3K4 methylation, in defined chromatin regions. Another source to obtain specificity is to combine H3K4me3 with other histone marks. This may allow for the recruitment of a combination of readers or single readers that possess more than one binding site for distinct marks and thus can read specific combinations of histone modifications. It is important to understand how H3K4 methylation relates to other histone marks, as these might enhance or restrict the positioning and/or the function of H3K4me3 [9,132].

**Figure 5:**
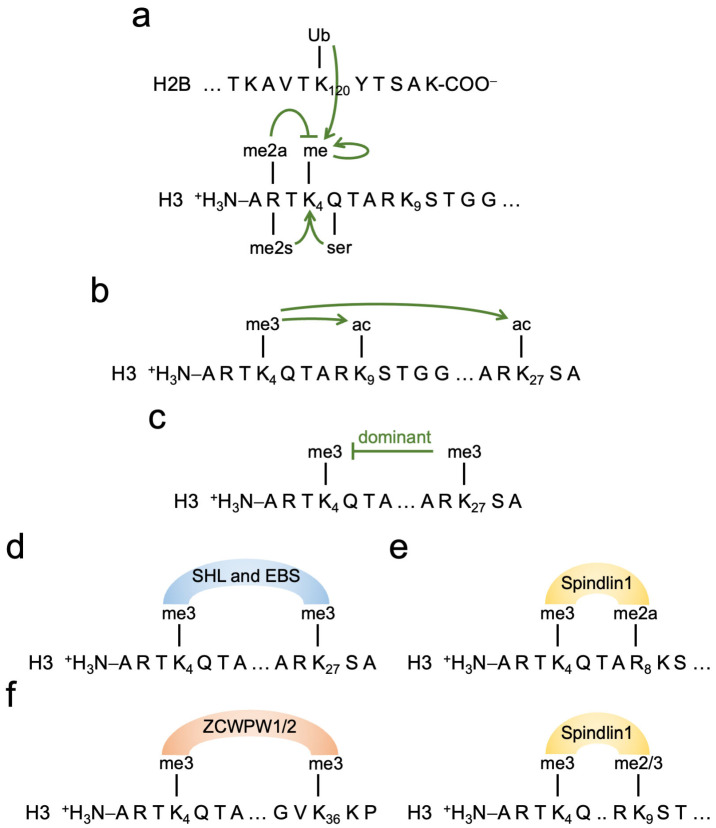
Interplay of H3K4me3 with other histone marks. (**a**) In addition to the recruitment of KMT2 complexes by TFs and other means, as summarized in the text, the ubiquitination of histone H2B lysine 120 provokes KMT2 complex recruitment and methylation of H3K4. Methylation of H3K4 is also stimulated by serotonylation of glutamine 5 of histone H3 (H3Q5ser) and by symmetrical di-methylation of arginine 2 (H3R2me2s) of histone H3. In contrast, asymmetrical R2 di-methylation (H3R2me2a) of histone H3 interferes with KMT2-dependent methylation of H3K4. (**b**) H3K4me3 and H3K4me1 (the latter is not shown) recruit complexes with HAT activity, modifying different lysines, including H3K9 and H3K27. This contributes to chromatin opening. (**c**) The modification of promoters with both H3K4me3 and H3K27me3 is referred to as bivalent chromatin. Such promoters are accessible but typically not transcribed or only at very low levels. Thus, it appears that in most situations, the repressing H3K27me3 mark is dominant over the positive H3K4me3 mark. (**d**–**f**) Combinations of histone marks are most likely important in order to determine specificity and define functional outcome. Examples of proteins that can interact with different histone mark combinations are given. SHL and EBS are Arabidopsis proteins that recognize H3K4me3/H3K27me3, i.e., a positive and a repressing histone mark (**d**). Spindlin1 reads H3K4me3 in a combination with H3R8me2a or H3K9me2/3 (**e**). ZCWPW are proteins that read H3K4me3/H3K36me3 in the context of meiotic recombination (**f**). For further details see the text.

Early on, it was realized that H3K4 methylation is stimulated by ubiquitination of H2BK120 (H2BK123 in yeast), which is also associated with transcription (Figure 5a) [59,60]. RBBP5 binds to H2BK120Ub and can recruit KMT2 complexes [133,134,135]. Thus, this ubiquitination provides a site for KMT2 complex recruitment, in addition to those described above.

Both active promoters and enhancers are characterized by lysine acetylation, with a particularly strong correlation with H3K27ac and H3K9ac (Figure 5b). In general, acetylation is associated with open chromatin and acetylation readers such as Bromodomain-containing proteins are associated with chromatin remodelers [12,54,136]. H3K4me3 and H3K27ac can occur on the same nucleosome [137]. This close proximity may be important for readers with two spatially restricted binding domains for these modifications. H3K27ac, as well as several other acetylated sites, stimulate H3K4me3-dependent TAF3 binding in vitro [111], consistent with TAF1 binding to H4Kac [138]. Moreover, KMT2A-D bind to nucleosomes that carry H3K4me3 and are rich in acetylation, particularly on histone H3 [95]. This may be a mechanism supporting the propagation of H3K4 methylation both at promoters and enhancers.

Serotonylation of glutamine 5 (Q5ser) is another histone mark associated with activation of gene expression (Figure 5a) [139,140,141,142]. Many different H3K4me3 readers tolerate H3Q5ser, but binding of TAF3 is promoted [139]. H3Q5ser is also read by WDR5. Cells that express mutants of WDR5 that prevent binding to H3Q5ser show reduced overall H3K4me3, indicating that the WDR5-H3Q5ser interaction contributes to maintaining H3K4me3 [143]. Importantly, TAF3 binding to H3Q5ser is independent of the methylation status of H3K4 [139], providing a possible explanation as to why the loss of H3K4me3 upon RBBP5 knockdown does not affect TFIID binding [43]. This suggests that H3Q5ser might be an alternative histone mark to H3K4me3 to support PIC loading and transcription.

Promoters that are marked with H3K4me3 and H3K27me3, the latter being a repressive mark catalyzed by the polycomb repressor complex PRC2, are silent and referred to as bivalent [144,145,146]. H3K4me3/H3K27me3 is found in stem and tumor cells. It is thought that by removing H3K27me3 a gene can be rapidly activated, while demethylating H3K4 results in further silencing. It appears that H3K27me3 is dominant over H3K4me3 (Figure 5c). H3K27me3 can be read by BAH domains. Interestingly, the SHL and EBS proteins in Arabidopsis, which contain PHD and BAH domains, bind to both H3K4me3 and H3K27me3 and regulate flowering (Figure 5d) [147,148]. The two proteins cooperate in switching the active chromatin state of the floral repressor FLC to a polycomb repressed state [147]. Although the mechanistic details are not fully understood, it is an example of proteins that read both an activating and a repressing mark. It will be interesting to see whether other readers exist that combine positive and repressive marks and what the consequences are. The important interplay of H3K4me3 and H3K27me3 is further documented by the ability of KMT2 complexes to recruit KDM6 enzymes, such as UTX, which demethylate H3K27me3 (Figure 4) [149,150,151,152,153]. This would then allow the acetylation of H3K27, possibly in response to acetyltransferases recruited by H3K4me3, to create a transcription-permissive environment.

Additional neighboring amino acids of H3K4 contribute to regulation. TAF3 binding is inhibited by phosphorylation of threonine 3 (H3T3P), a site modified by Harpin possibly linked to the inhibition of transcription in mitosis, while maintaining H3K4me3 may serve as memory to reactivate genes in early G1 [154,155,156]. A more recent study indicates that H3K4 methylation readers are displaced in mitosis also in the absence of Harpin, suggesting that transcriptional down-regulation is achieved by other means than H3T3P [157]. Threonine 6 of histone H3 (H3T6P) is phosphorylated by PKCβ [158]. This modification prevents the binding of KDM1A and may stabilize H3K4 methylation and thus possibly regulate gene expression. This might be particularly relevant at enhancers, because KDM1A preferentially demethylates H3K4me1 and me2. It has been suggested that T3P and/or T6P, together with K4 methylation, might form a phospho/methyl switch. However, this would require that the marks are on the same histone tail, for which evidence is presently lacking (see discussion in [157,159]). 

The asymmetric di-methylation of histone H3 arginine 2 (H3R2me2a) interferes with H3K4 methylation in cis (Figure 5a) [160,161,162]. In cells, the loss of H3R2me2a promotes KMT2 complex binding and gene expression [163]. In contrast, the symmetric H3R2me2s is associated with H3K4me3 at active promoters as well as at sites of recombination [164]. Moreover, the combination of H3K4me3 with either R8me2a or K9me3/2 is read by Spindlin1, stimulating the transcription of rRNA genes (Figure 5e) [165,166,167,168]. These examples document the interplay of H3K4 methylation with other histone marks. Further complexity can be envisaged when more than two histone marks are considered, including marks that so far have not been studied in detail.

During recombination in meiosis and antibody and T cell receptor maturation, relevant chromatin regions are modified with H3K4me3/H3K36me3 (Figure 2) [19,36]. H3K36me3 is associated with transcribed regions, exons in particular, but not with promoters [169,170,171]. Thus, the close proximity of H3K4me3 and H3K36me3 in chromatin is not linked to transcription. The combination of H3K4me3 and H3K36me3 is read by ZCWPW1 and its paralog ZCWPW2 (Figure 5f), which are part of a process that involves meiotic recombination-associated DNA double-strand breaks and repair by homologous recombination [19,35]. Interestingly, organisms that lack PRDM9 induce recombination at other sites that possess H3K4me3, for example, at promoters, as observed in *Prdm9* knockout mice [19]. Moreover, PRDM9-less organisms also lack ZCWPW1/2 readers [172]. H3K4me3 is important for V(D)J recombination, which involves the recruitment of the RAG complex to so-called recombination signal sequences [173]. The binding of RAG2 to H3K4me3 and of RAG1 to H3K36me3 stimulates recombination [37]. Thus, as for meiotic recombination, a combination of H3K4me3 and H3K36me3 appears to promote V(D)J recombination.

In order for recombination to take place, DNA double-strand breaks are required, and thus H3K4me3/H3K36me3 might also be linked to DNA repair. ASH1L, which can tri-methylate both H3K4 and H3K36 (Figure 2) [174,175,176,177], is indirectly recruited to certain forms of DNA damage, such as cyclobutane pyrimidine dimers (CPDs), and contributes to the nucleotide excision repair process [18,178]. The activity of ASH1L to modify both H3K4 and H3K36 is stimulated by MRG15, a protein that interacts with methylated H3K36 [179]. Thus, besides a role in transcription, H3K4me3 marks in combination with H3K36me3 are linked to recombination and DNA repair.

The observations discussed above provide evidence for the cooperative and antagonistic consequences of the combination of histone marks. It is likely that these are not exotic examples but the beginning of understanding how histone marks interact. In particular, multi-subunit complexes may contain more than one reader domain and thus are predicted to be suited to interpret combinatorial marks. Because of the multi-valency of the interactions of large complexes and chromatin, it is likely that not all binding sites for readers need to be present. In other words, H3K4me3 may be dispensable for the transcription of some genes, because compensatory, alternative mechanisms are in place to control RNAPII.

## 5. Manipulating H3K4 Methylation and Their Consequences on Gene Expression

The six KMT2 catalytic subunits in mammals are essential for organismal development and in part have redundant functions that make their characterization challenging [15,16,30]. Yeast has a single catalytic subunit, Set1, simplifying its analysis. Deletion of *set1* in the budding yeast *Saccharomyces cerevisiae* suggests that Set1 is not essential for short-term viability, but plays a role in different aspects of growth and development, and only few genes are deregulated [26,27,29,180,181,182]. Thus, H3K4 methylation seems not to be essential for viability. To support this, yeast mutants with H3K4 replaced by arginine or alanine (H3K4R or H3K4A, respectively), which cannot be methylated at position 4 of histone H3, show a slow growth phenotype, similar to *set1D*, as well as the deletion of other COMPASS subunits [26,29]. These observations are complemented by a study in *Drosophila melanogaster*. Flies possess three catalytic subunits, dSet1, Trithorax, and Trithorax-related, the orthologues of SET1A/B, MLL1/2, and MLL3/4, respectively (Table 1). Cells in which all copies of histone H3 encoding genes have been replaced with mutant versions that lack lysine 4, i.e., H3K4R or H3K4A, proliferate, albeit slower than wildtype cells, and respond to signaling by activating target gene expression [183]. Together, these findings provide strong arguments to suggest that H3K4 methylation is not essential for gene transcription.

The knockouts of genes that encode core components of KMT2 complexes, i.e., WDR5, ASH2L, and DPY30, are lethal and typically many hundreds to thousands of genes are deregulated [58,184,185,186,187,188,189]. This is paralleled by a substantial decrease in H3K4me3. Because the consequences of the knockouts of *Dpy30* and *Ash2l* in the hematopoietic system of mice are very similar [187,188], it seems likely that the reduced activity or inactivation of KMT2 complexes is the main driver of the observed phenotypes.

The finding that H3K4 methylation is not essential in the model systems described above seems at odds with the importance of the different KMT2 complex subunits. In the murine system, the knockout of an individual subunit may result in a specific defect in certain tissues, such as, for example, the loss of MLL1 in hematopoiesis and in neurogenesis [190,191], which define essential functions for the expression of specific genes, thereby potentially affecting the whole organism. Another possibility is that other KMT2 substrates exist that are relevant for the observed phenotypes [39]. Indeed, a few non-histone substrates of KMT2 enzymes have been identified. These include the transcription factor YAP, a key effector of the Hippo signal transduction pathway [192]. This pathway is protumorigenic, and SET1A-mediated mono-methylation of YAP promotes its nuclear localization, thereby stimulating transcription. Another is the transcription factor and tumor suppressor p53, although this has only been demonstrated in vitro [193]. In addition to these two transcription factors, the heat shock protein HSP70 is a SET1A substrate and its methylation stimulates its nuclear localization, binding to and activating Aurora A kinase [194]. Methylated HSP70 has protumorigenic activities, possibly by regulating Aurora A kinase activity, a key player in cell cycle regulation both in normal and cancer cells [195]. This is likely the proverbial tip of the iceberg, and additional substrates may surface with more extensive screens. In this respect, studies with SET8/KMT5A are of interest. This enzyme mono-methylates H4K20, which is associated with multiple chromatin-associated functions [196]. The changes in gene expression observed upon loss of SET8 are distinct from mutating H4K20 to arginine, suggesting that other SET8 substrates are important [197,198]. Indeed, other substrates have been described, although their functional relevance for gene expression is not well understood [196,199]. Thus, it will be interesting to define whether additional non-histone substrates of KMT2 complexes can be defined and whether these contribute to the observed effects on gene expression and cell proliferation as well as the pathologies associated with these methyltransferase complexes [4,200,201,202].

## 6. PROTAC Systems to Rapidly Control KMT2 Complex Functions

Knockout or siRNA-mediated knockdown studies have been very important to address the functions, in particular, the loss of function, of multiple proteins. A disadvantage of such studies is that the decrease in protein levels can be slow and, additionally, in the case of knockdown experiments, the loss of relevant proteins is typically only partial. The slowness of these systems may limit how findings can be interpretated, as secondary consequences may be difficult to distinguish from primary effects. Also, thresholds of protein levels may impact functional consequences. Inhibitors can overcome some of these limitations, as they can rapidly inactivate catalytic functions of enzymes or interfere with protein–protein interactions. But inhibiting an enzyme may not be identical to removing the protein, and not all proteins of interest are enzymes. Moreover, few small molecules are available to control protein–protein interactions and complex formations. It is worth pointing out that various attempts have been and are being made to interfere with KMT2 complex functions due to their association with disease [4,15,16,17]. One focus is on KMT2A/MLL1, as this catalytic subunit is well described in cancer. This includes targeting the interaction of Menin with KMT2A, SAM mimetics, and targeting WDR5 [203,204,205]. These efforts are mainly driven for clinical applications, but their selectivity and efficacy appear to be challenging [45,206,207,208]. One might expect that WDR5 degraders affect all KMT2 complexes, but it has been argued that KMT2 complexes are not uniformly inhibited, as one might expect, but that KMT2A containing complexes are particularly sensitive [209]. Altogether, these findings are highly relevant in view of disease treatment but are limited in addressing the mechanisms associated with H3K4 methylation, as not all complexes can be manipulated in parallel.

**Figure 6:**
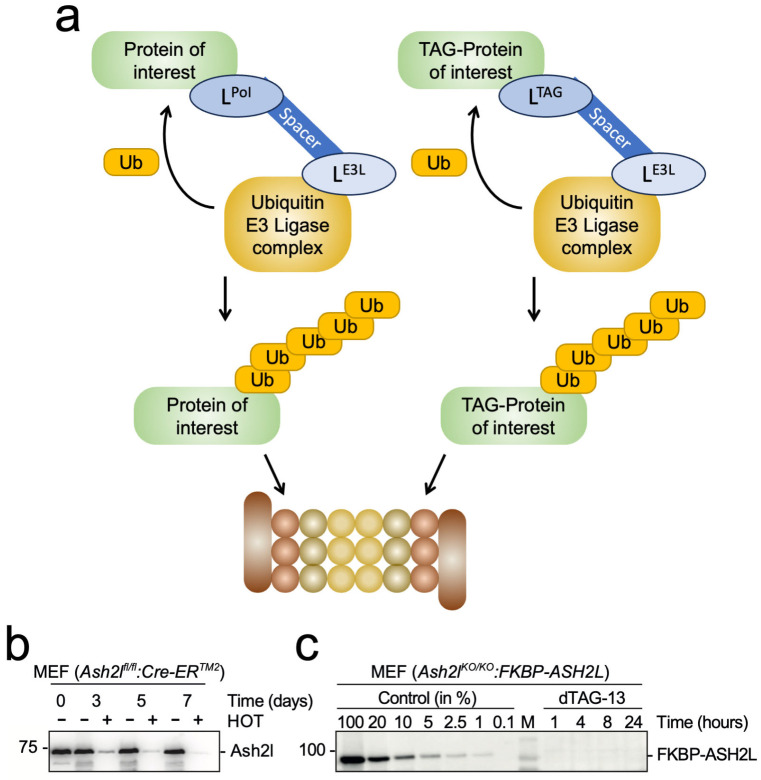
Proteolysis targeting chimera (PROTAC) for the rapid degradation of proteins of interest (PoIs). (**a**) Schematic of PROTAC systems. Ligands (L) are used to interact either directly with a protein of interest (L^PoI^, **left panel**) or target a tagged version of the PoI (L^TAG^, **right panel**). Optimally, this results in the poly-ubiquitination of the PoI and its subsequent proteasomal degradation. (**b**) Ash2l expression is shown in mouse embryo cells (MEF) with a floxed *Ash2l* locus. Upon deletion of the *Ash2l* loci in response to 4-hydroxytamoxifen (HOT) treatment, protein expression is measured over several days. The data are taken from [189]. (**c**) MEF cells with deleted *Ash2l* loci express a FKBP-ASH2L fusion protein. Upon adding the bi-functional PROTAC dTAG-13, which binds to both FKBP and the Cereblon subunit of a Ubiquitin E3 ligase complex, FKBP-ASH2L is poly-ubiquitinated and degraded in the proteasome. The data are taken from [51].

When considering KMT2 complexes and H3K4 methylation, a relevant question would be, for example, how much of H3K4me3 is functionally relevant? Are there thresholds of H3K4me3 levels that support some but not other functions, possibly dependent on other histone marks? Inhibitors of all KMT2 complexes, which might overcome some of the limitations discussed above, have not been reported. However, the development of PROTAC systems to rapidly degrade selective targets has become an alternative approach (Figure 6a) [210]. In recent studies, PROTACs have been used to target KMT2 complex subunits, causing their rapid and substantial decrease [43,44,51,52]. Of note, these are knockdown systems. The proteins are made but very rapidly degraded to potentially very low levels. For example, in the case of ASH2L less than 1% of the protein was left over after 1 h of PROTAC treatment (Figure 6b,c) [51]. Moreover, in the studies of KMT2 complex subunits, all proteins are fusion proteins, to allow for targeting. This requires validation that the fusions are functionally comparable to their wildtype counterparts.

Of particular importance are the studies addressing WRAD subunits, because their loss affects all KMT2 complexes. Indeed, targeting RBBP5, ASH2L, or DPY30 with PROTACs results in a rapid decrease in H3K4me3 at promoters [43,44,51]. The dynamics of H3K4 methylation loss upon knockdown of RBBP5 and DPY30 are very similar [43,44], suggesting that DPY30, which contributes little to the in vitro methyltransferase activity of KMT2 complexes [211,212], is equally important in cells, consistent also with the knockout studies mentioned above [187,188,213]. However, some differences are worth pointing out. For example, whereas the loss of DPY30 or RBBP5 reduced cell proliferation of mESCs by roughly a third [44], MEF cells ceased proliferation upon ASH2L loss [51]. It is interesting that mESCs proliferate in the absence of any substantial H3K4me3, while MEF cells cannot, a reminder of the experiments in flies and yeast discussed above [26,27,29,181,183], although these differences remain unexplained.

Despite this difference in cell proliferation, the decline in H3K4me3 and the deregulation of genes were considerably faster in the mESCs compared to the MEF cells [43,44,51]. Of note is also that RNAs were not only down- but also up-regulated, already noted upon *Ash2l* KO in the hematopoietic system [188]. The latter might be due to secondary effects, which would be expected to be less prevalent when using PROTACs. Similarly, the PROTAC-mediated loss of SET1A/B results in both down- and up-regulated RNAs, with many deregulated by 2 h, which makes indirect effects less likely [52]. Nevertheless, the strong effects that already occur after two hours are surprising, as the decrease in H3K4me3 at promoters is small. Possibilities are that the SET1A/B loss may influence RNA stability or reactivate repressed genes. Alternatively, some of the effects of SET1A/B could be independent of H3K4 methylation, an option that is not unlikely considering the large multi-domain organization of these proteins but also of the other KMT2 catalytic subunits (Figure 1).

These experiments determine the final products, which might be misleading. Measuring ongoing transcription may provide a more direct picture of the consequences of losing functional KMT2 complexes and H3K4me3. Indeed, reduced transcription was measured in mESCs upon depletion of either DPY30 or RBBP5 as early as 2 h after PROTAC treatment [43,44]. This being directly linked to H3K4me3 is supported by analyzing cells with a deletion of *Kdm5a/b* or inhibition of the enzymes, which stabilizes H3K4me3; as a result, reduced transcription was delayed considerably. In MEF cells, the effects were slower, as only a few genes were affected upon the loss of ASH2L in the first 8 h, consistent with a slow decrease in H3K4me3 [51]. Together, these studies suggest a role of H3K4me3 for RNAPII activity at least at some genes.

## 7. Is H3K4me3 Sufficient to Activate Gene Expression?

Although this seems like an important question, it cannot really be answered. Two experiments are relevant to mention here. Targeting a SET1A fusion protein to an endogenous reporter gene is sufficient to stimulate transcription, an effect that is independent of SET1A catalytic activity [52]. Targeting ASH2L to an endogenous gene locus enhances transcription dependent on both the SPRY and SDI domains, which are necessary for KMT2 complex formation [51]. This set up does not provide information regarding KMT2-dependent methyltransferase activity but suggests that positioning KMT2 complexes to a specific promoter is sufficient to enhance transcription. In both experimental setups, the subunits that are directed to specific chromatin sites can undergo multiple interactions and result in the assembly of KMT2 complexes. In turn, various additional interactions may form, including with cofactors that promote transcription.

Considering the functional consequences of H3K4 methylation by recruiting both remodeling complexes and cofactors that modify histones, for example, by acetylation, it becomes obvious that H3K4 methylation cannot be analyzed in isolation. By definition, additional chromatin modifications are a consequence of this mark. Nevertheless, the information available suggests that H3K4 methylation is important in many circumstances to participate in the control of gene expression.

Another aspect worth considering is that a substantial number of genes are activated upon the loss of WRAD components. The promoters of some of these genes are not marked with detectable levels of H3K4me3, suggesting that other modifications exist that can define an active promoter and possibly compensate for the lack of H3K4 methylation [43,51]. One such modification could be H3Q5ser, as discussed above, because of its ability to recruit TFIID and thus potentially the RNAPII complex [139]. But TFIID binding is unlikely to be sufficient to activate gene expression. Most certainly, combinatorial effects are required that bring various activities to enhancers and promoters to promote gene expression, both by direct interactions or by forming condensates that accommodate multiple factors. From the limited amount of information we have at present, the key role of H3K4 methylation stands out because so many readers have been identified that modulate and amplify the signals generated by H3K4 methylation.

## 8. Does H3K4 Methylation Have a Role in Chromatin Organization?

The findings summarized suggest that H3K4me3 influences chromatin accessibility, which appears to contribute to transcription, recombination, and DNA repair. However, this discussion is complex. On the one hand, a strong correlation between active enhancers and promoters and their modification by H3K4me1 and H3K4me3, respectively, is observed. But on the other, it has been argued that decisive mechanistic evidence on how these modifications affect promoter function has not been sufficiently defined [131]. Other interesting observations complicate the understanding of the role of H3K4me3, which is enriched near TADs and sub-TADs [18,214,215]. Several studies suggest that H3K4 methylation, including broad H3K4me3 regions, is associated with chromatin organization [216,217,218,219]. An interplay between H3K4me3 and chromatin organization has also been suggested by a recent study that demonstrates a PHF2-mediated link between H3K4me3 marked promoters and cohesin [220]. Cohesin, a multi-subunit complex that belongs to a small family of eukaryotic SMC complexes, is involved in loop and TAD formation, together with CTCF [215,221,222]. The interaction of cohesin with promoters is important to limit the size of repressive B compartments [220,223]. Moreover, many TAD boundaries that contain CTCF are in the close vicinity of promoters, and thus potentially of H3K4me3-modified regions [224,225]. In addition to cohesin, condensin complexes, another SMC family member, are also linked to H3K4me3. Condensin II binds to H3K4me3 through the CAP-D3/NCAPD3 complex and supports gene expression near TADs [226]. These findings support a close interplay between H3K4me3, gene transcription, and chromatin organization. Whether H3K4me3 is located at or close to TADs because of close-by promoter(s) or whether this histone mark has a function beyond transcription, possibly in higher order chromatin organization, needs further evaluation.

The broad deregulation of gene expression observed upon the loss of WRAD core components might be affected by altered chromatin organization, for example, by affecting TADs, in addition to local effects on promoters [43,44,51]. CTCF ChIP-seq experiments in response to ASH2L depletion revealed some repositioning of CTCF both at TAD boundaries and promoters, supporting the link between H3K4me3 and chromatin organization [58]. How decisive such alterations are, however, is not well understood.

## 9. Conclusions

The available information suggests that H3K4me3 plays important roles in transcription as well as in recombination and DNA repair. For transcription, the model suggests that KMT2 complexes and H3K4 methylation participate in the control of chromatin remodeling and opening, the recruitment of writers and erasers of histone marks, RNAPII complex recruitment, and reinitiation of transcription. This requires that the different KMT2 complexes are recruited to specific chromatin regions. Indeed, multiple mechanisms have been identified to position KMT2 complexes, which include sequence-specific TFs, non-methylated CpG dinucleotides prevalent at CGI promoters, and RNAPII. These interactions are consistent with the strong association of H3K4me1 with enhancers and H3K4me3 with promoters. Moreover, the multiple readers that have been identified of the different H3K4 methylated states and their associated functions, such as ATPase-dependent chromatin remodelers and enzymes that post-translationally modify core histones and thus modulate accessibility and offer binding sites for additional factors, provide mechanistic insight into the consequences for gene transcription. Together, these findings provide functional evidence that explains the important role of H3K4 methylation in promoting transcription.

Because all the components of the KMT2/COMPASS-like complexes are essential during development and/or during tissue homeostasis, it seems self-evident that the methylation of H3K4 is also essential. However, this is not the case, as at least some cell types are able to proliferate and respond to signals in the absence of H3K4 methylation. One option that needs to be considered is that substrates beyond H3K4 exist that have important functions and are regulated by KMT2 complexes. At present, we only have limited information regarding non-histone substrates, and future studies should be designed to screen for such alternative substrates.

An important area of research is to understand how H3K4 methylation interacts with other histone marks. A number of important findings have been made that suggest that H3K4 methylation is, on the one hand, influenced by some histone PTMs, and on the other hand, affects various other marks. Considering the large number of PTMs that have been described for core histones, it is likely that many additional interactions will be discovered in the coming years. It will be important to define the combinations of histone marks that define transcription, both positively and negatively. A question that remains open is whether all combinations of marks that stimulate transcription require H3K4me3 at promoters or whether this modification can be compensated for. Another question that is raised is how combinations of histone marks are read. Indeed, some proteins have been defined that are capable of binding to combinations of marks. We would expect that this is an area that will grow considerably in the years to come, as there is still very limited information available, particularly considering protein complexes and condensates that may bring together multiple readers.

We think it is important to note that if H3K4me3 is indeed critical for the efficient and accurate transcription of many genes, multiple processes in the cell will be dependent on this mark. Thus, those genes critical for a given process that are deregulated first may well dominate the observed cellular phenotypes. It is therefore not surprising that many reports describe specific functional consequences upon manipulating KMT2 complexes, sometimes dependent on the deregulation of a single gene. We think it is likely that at least in some instances, such conclusions are oversimplified.

Finally, we note that considerable progress has been made in defining the spatial and temporal loading of KMT2 complexes to chromatin sites such as promoters and enhancers. Most likely, the functional consequences will also be defined by mechanisms that control the assembly and the catalytic activities of these complexes. Many PTMs have been identified on the various KMT2 complex subunits as well as on the demethylases, for example, by unbiased mass spectrometry analyses (see, e.g., https://www.phosphosite.org). Most likely, not all of these will be of functional relevance; nevertheless, the complexity is staggering but poorly understood. For a more complete understanding of how H3K4 methylation is controlled by both writers and erasers, it will be necessary to define the relevant signaling pathways and regulatory networks underlying this mark. This is an important area of future research.

## Figures and Tables

**Table 1:** Components of KMT2 complexes.

	MTase (Writers)	WRAD Subunits (Common to All Complexes)				Additional Subunits	Activity (Main)
Yeast	ySet1	Cps30 (Swd3)	Cps50 (Swd1)	Cps60 (Bre2)	Cps25 (Sdc1)	Cps35 (Swd2, Wdr82) Cps40 (Spp1, Cfp1)	H3K4me1-3
Drosophila melanogaster	Trithorax	WDS	RBBP5	ASH2	DPY30	HCF1/2	H3K4me(1-)3
	Trithorax related					PTIP PA1 NCOA6 UTX	H3K4me1
	dSet1					WDR82 CXXC1 HCF1/2	H3K4me(1-)3
Mammals	MLL1/ KMT2A	WDR5	RBBP5	ASH2L	DPY30	HCF1/2 Menin PSIP1 (LEDGF)	H3K4me(1-)3
	MLL2/ KMT2B						
	MLL3/ KMT2C					PTIP PA1 NCOA6 UTX	H3K4me1
	MLL4/ KMT2D						
	SET1A/ SETD1A/ KMT2F					WDR82 CXXC1 HCF1	H3K4me(1-)3
	SET1B/ SETD1B/ KMT2G						

## Data Availability

No new data were created or analyzed in this study. Data sharing is not applicable.

## Supplementary Materials

The following supporting information can be downloaded at: https://www.mdpi.com/article/10.3390/molecules30204075/s1, Table S1: Interaction of transcription factors with KMT2 complexes.

## Abbreviations

ac	Acetylation
ATAC-seq	Assay for Transposase accessible chromatin with high-throughput sequencing
BAH	Bromo-adjacent homology domain
CFP1	CxxC zinc finger protein 1
CGI	CpG island
ChIP-seq	Chromatin immunoprecipitation with high-throughput sequencing
COMPASS	Complex of proteins associated with SET1
CTD	C-terminal domain of RNAPII
FAD	Flavin adenine dinucleotide
H3K4	Histone H3 lysine 4
IDR	Intrinsically disordered region
ING	Inhibitor of growth protein
KDM	Lysine demethylase
KMT	Lysine methyltransferase
me1-3	Mono-, di- and tri-methylation
Rme2s	Symmetric di-methylation of arginine
Rme2a	Asymmetric di-methylation of arginine
MLL	Mixed lineage leukemia
NDR	Nucleosome-depleted regions
NURF	Nucleosome remodeling factor
PIC	RNAPII pre-initiation complex
PoI	Protein of interest
PROTAC	Proteolysis targeting chimera
PTM	Post-translational modification
RNAPII	DNA-dependent RNA polymerase II
S2P/S5P/T3P/T6P	Phosphorylated serine 2 or 5 or threonine 3 or 5
SAGA	Spt-Ada-Gcn5 acetyltransferase complex
SAM	S-adenosyl-L-methionine
SET	Su(var)3-9, Enhancer-of-zeste and Trithorax domain
SMC	Structural maintenance of chromosome complex
SWI/SNF	SWItch/Sucrose Non-Fermentable remodeling complexes
TAD	Topologically associated domains
TAF	TATA-box binding protein associated factor
TF	Transcription factor
