# Supplementary material for: Role of Histone H3 Lysine 4 Methylation in Chromatin Biology
Source: Molecules. 2025 Oct 14;30(20):4075. doi: 10.3390/molecules30204075 (PMC12565824; doi:10.3390/molecules30204075)
Supplement: Supplementary file 1 [file molecules-30-04075-s001.zip › molecules-3855763-supplementary.pdf]

Role of Histone H3 Lysine 4 Methylation in Chromatin Biology

Bernhard Lüscher, Philip Bussmann and Janina Müller

Supplementary Table S1

Interaction of transcription factors with KMT2 complexes

Trans-cription factor	WDR5	RBBP5	ASH2L	DPY30	MLL1 / KMT2A	MLL2 / KMT2B	MLL4 / KMT2D	SET1A / KMT2F	Pioneer*	References
ANCCA										[1]
AP2∂			YES							[2]
BRAC	0	YES	0	0	0	0	YES	0		
CPEB1	0	0	0	0	0	0	0	0		
CREB1	0	0	0	0	0	0	0	0		
E2F										[1, 3]
ELF1	0	YES	YES	0	YES	YES	0	YES		
ELF2	0	YES	YES	0	YES	YES	0	YES		
ELF3	0	0	0	0	0	0	0	0		
ELF4	0	YES	YES	0	0	YES	YES	YES		
ELF5	0	0	0	0	0	0	YES	0		
ELK3	0	YES	0	0	0	YES	0	YES		
ELK4	0	0	0	0	0	0	0	0		
ERα									(YES)	[4]
ERG	0	0	0	0	0	0	YES	0		
ESR1	0	0	0	0	0	0	0	0		
ETS1	0	0	0	0	0	0	YES	0		
ETV3	0	0	0	0	0	0	0	0		
ETV4	0	0	0	0	0	0	YES	0		
ETV5	0	0	0	0	0	0	YES	0		
ETV6	0	0	0	0	0	0	0	0		
ETV7	0	0	0	0	0	0	0	0		
FEV	0	0	0	0	YES	YES	YES	YES		
FOS	0	YES	YES	0	0	0	YES	0		
FOXA1									YES	[5]
FOXI1	0	0	0	0	0	0	YES	0		
FOXL1	0	0	0	0	0	0	YES	0		
FOXQ1	0	0	0	0	0	0	0	0		
FXR										[6]
GATA1	0	0	0	0	0	0	YES	0		

GATA2	0	YES	0	0	0	0	YES	0	YES	
GATA3	0	0	0	0	0	0	YES	0	YES	
GCM1	0	0	0	0	0	0	YES	0		
GLI2	0	0	0	0	0	0	0	0		
GLI3	0	0	0	0	0	0	0	0		
HEN1	0	0	0	0	YES	0	YES	0		
HME1	0	0	0	0	YES	0	YES	0		
HNF1a	0	0	0	0	0	0	YES	0		
HNF1B	0	YES	0	0	0	0	YES	0		
HNF4a	YES	YES	YES	0	0	YES	YES	YES		
IRF1	0	0	0	0	0	0	0	0		
IRF4	0	0	0	0	0	0	YES	0		
IRF5	0	0	0	0	0	0	0	0		
IRF8	0	0	0	0	0	0	YES	0		
IRF9	0	0	0	0	0	0	0	0		
ISX	0	0	0	0	0	0	0	0		
KLF3	YES	0	0	0	YES	YES	YES	0		
KLF4	0	0	0	0	0	0	YES	0	YES	
KLF5	0	YES	0	0	0	0	YES	0		
KLF6	0	0	0	0	0	0	0	0		
KLF8	0	0	0	0	0	YES	YES	YES		
KLF9	0	0	0	0	0	0	0	0		
KLF10	0	0	0	0	0	0	0	0		
KLF12	YES	0	0	0	YES	YES	0	YES		
KLF15	0	0	0	0	0	0	YES	0		
KLF16	0	0	0	0	0	YES	YES	0		
LHX1	0	0	0	0	0	0	YES	0		
LHX2	0	YES	YES	0	0	0	YES	0		
LHX3	0	YES	0	0	0	0	YES	0		
LHX4	0	0	0	0	0	0	YES	0		
LHX6	0	0	0	0	0	0	0	0		
LHX8	0	0	0	0	0	0	YES	0		
MAFA/B										[7]
MEF2A	0	0	0	0	0	0	0	0		
MEF2D										[8]
MSX1			YES	YES						[9]
MYB	0	0	0	0	0	9	YES	YES		[10]
MYC	YES	YES	YES	0	YES	YES	0	YES		[11, 12]
MYOD1	0	YES	YES	0	0	0	YES	0	YES	
NANOG										[8]
NFAC3	0	0	0	0	0	0	0	0		
NFAC4	0	0	0	0	0	0	0	0		
NF-E2										[13, 14]
NFIA	0	0	0	YES	0	0	YES	0		
NFIB	0	0	0	YES	0	0	YES	0		

NFIC	0	0	0	YES	0	0	YES	0		
NFIX	0	YES	YES	0	YES	YES	YES	YES		
NFKB1	0	0	0	0	0	0	0	0		
NFYA			YES							[15]
NFYC	0	0	0	0	YES	0	0	YES		
OCT4	YES		YES						YES	[8, 16, 17]
PAX2	0	0	0	0	0	0	YES	0		[18, 19]
PAX5										[20, 21]
PAX6	0	YES	YES	0	YES	YES	YES	YES	YES	[22]
PAX7	0	0	0	0	0	0	YES	0	YES	[23]
PAX8	0	YES	0	0	0	0	YES	0		
PAX9	0	0	0	0	0	0	YES	0		
PPARG	0	0	0	0	0	0	YES	0		
PRDM1	0	0	0	0	0	0	0	0		
RREB1	0	0	0	0	0	0	0	0		
SMAD5	0	0	0	0	0	0	0	0		
SOX2	YES	YES	YES	0	0	0	YES	0	YES	[8, 17]
SOX4	0	0	0	0	0	0	0	0		
SOX5	0	0	0	0	0	0	YES	0		
SOX6	0	0	0	0	0	0	0	0		
SOX9	0	0	0	0	0	0	YES	0	YES	
SOX10	0	0	0	0	0	0	YES	0		
SOX15	0	0	0	0	0	0	YES	0		
SOX17	0	0	YES	0	0	0	YES	0		
SP1	0	0	0	0	0	0	0	0		
SP7	0	0	YES	0	0	YES	YES	0		
SPZ1	0	0	0	0	0	0	0	0		
STAT1	0	0	0	0	0	0	0	0		
STAT3	0	0	0	0	0	0	0	0		
STAT4	0	0	0	0	0	0	YES	0		
TAL1	0	0	0	0	0	0	0	0		
TBR1	0	YES	0	0	0	0	YES	0		
TEAD1	0	0	0	0	0	0	YES	0		
TEAD2	0	0	0	0	0	0	0	0		
TEAD3	0	0	0	0	0	0	0	0		
TFAP2a			YES	YES						[9]
TLX1	0	0	0	0	0	0	YES	0		
TLX2	0	0	0	0	0	0	YES	0		
TLX3	YES	0	0	0	0	0	YES	0		
TP53									YES	[24]
TYY1	YES	YES	YES	0	YES	YES	0	YES		
USF1										[25]
VSX1	0	0	0	0	0	0	0	0		

Green and blue: Interactors of the indicated 109 transcription factors using BioID/MS. The core subunits (green) and the catalytic subunits (blue) of KMT2 complexes are indicated that are biotinylated (no information for KMT2C and SET1B). YES indicates that a signal was detected (correct numbers of the mass spectrometry data can be found in the original publication). Modified from Göös et al. [26]. For more details see the text.
Yellow: Transcription factors shown to associate with KMT2 complexes, in addition to those reported in Göös et al. [26].
* Pioneer transcription factors as summarized before [27-30].
If none of the components is indicated then the direct interaction partner is not known or is one of the other subunits as summarized in Table 1.

References

1. Tyagi, S., A. L. Chabes, J. Wysocka, and W. Herr. "E2f Activation of S Phase Promoters Via Association with Hcf-1 and the Mll Family of Histone H3k4 Methyltransferases." *Mol Cell* 27, no. 1 (2007): 107-19.
2. Tan, C. C., K. V. Sindhu, S. Li, H. Nishio, J. Z. Stoller, K. Oishi, S. Puttreddy, T. J. Lee, J. A. Epstein, M. J. Walsh, and B. D. Gelb. "Transcription Factor Ap2delta Associates with Ash2l and Alr, a Trithorax Family Histone Methyltransferase, to Activate Hoxc8 Transcription." *Proc Natl Acad Sci U S A* 105, no. 21 (2008): 7472-7.
3. Takeda, S., D. Y. Chen, T. D. Westergard, J. K. Fisher, J. A. Rubens, S. Sasagawa, J. T. Kan, S. J. Korsmeyer, E. H. Cheng, and J. J. Hsieh. "Proteolysis of Mll Family Proteins Is Essential for Taspase1-Orchestrated Cell Cycle Progression." *Genes Dev* 20, no. 17 (2006): 2397-409.
4. Mo, R., S. M. Rao, and Y. J. Zhu. "Identification of the Mll2 Complex as a Coactivator for Estrogen Receptor Alpha." *J Biol Chem* 281, no. 23 (2006): 15714-20.
5. Jozwik, K. M., I. Chernukhin, A. A. Serandour, S. Nagarajan, and J. S. Carroll. "Foxa1 Directs H3k4 Monomethylation at Enhancers Via Recruitment of the Methyltransferase Mll3." *Cell Rep* 17, no. 10 (2016): 2715-23.
6. Ananthanarayanan, M., Y. Li, S. Surapureddi, N. Balasubramaniyan, J. Ahn, J. A. Goldstein, and F. J. Suchy. "Histone H3k4 Trimethylation by Mll3 as Part of Ascom Complex Is Critical for Nr Activation of Bile Acid Transporter Genes and Is Downregulated in Cholestasis." *Am J Physiol Gastrointest Liver Physiol* 300, no. 5 (2011): G771-81.
7. Scoville, D. W., H. A. Cyphert, L. Liao, J. Xu, A. Reynolds, S. Guo, and R. Stein. "Mll3 and Mll4 Methyltransferases Bind to the Mafa and Mafb Transcription Factors to Regulate Islet Beta-Cell Function." *Diabetes* 64, no. 11 (2015): 3772-83.
8. Ang, Y. S., S. Y. Tsai, D. F. Lee, J. Monk, J. Su, K. Ratnakumar, J. Ding, Y. Ge, H. Darr, B. Chang, J. Wang, M. Rendl, E. Bernstein, C. Schaniel, and I. R. Lemischka. "Wdr5 Mediates Self-Renewal and Reprogramming Via the Embryonic Stem Cell Core Transcriptional Network." *Cell* 145, no. 2 (2011): 183-97.
9. Mohammadparast, S., and C. Chang. "Ash2l, an Obligatory Component of H3k4 Methylation Complexes, Regulates Neural Crest Development." *Developmental biology* 492 (2022): 14-24.

10. Jin, S., H. Zhao, Y. Yi, Y. Nakata, A. Kalota, and A. M. Gewirtz. "C-Myb Binds Mll through Menin in Human Leukemia Cells and Is an Important Driver of Mll-Associated Leukemogenesis." *J Clin Invest* 120, no. 2 (2010): 593-606.
11. Ullius, A., J. Luscher-Firzlaff, I. G. Costa, G. Walsemann, A. H. Forst, E. G. Gusmao, K. Kapelle, H. Kleine, E. Kremmer, J. Vervoorts, and B. Luscher. "The Interaction of Myc with the Trithorax Protein Ash2l Promotes Gene Transcription by Regulating H3k27 Modification." *Nucleic acids research* 42, no. 11 (2014): 6901-20.
12. Thomas, L. R., Q. Wang, B. C. Grieb, J. Phan, A. M. Foshage, Q. Sun, E. T. Olejniczak, T. Clark, S. Dey, S. Lorey, B. Alicie, G. C. Howard, B. Cawthon, K. C. Ess, C. M. Eischen, Z. Zhao, S. W. Fesik, and W. P. Tansey. "Interaction with Wdr5 Promotes Target Gene Recognition and Tumorigenesis by Myc." *Mol Cell* 58, no. 3 (2015): 440-52.
13. Kim, A., S. H. Song, M. Brand, and A. Dean. "Nucleosome and Transcription Activator Antagonism at Human Beta-Globin Locus Control Region Dnase I Hypersensitive Sites." *Nucleic Acids Res* 35, no. 17 (2007): 5831-8.
14. Demers, C., C. P. Chaturvedi, J. A. Ranish, G. Juban, P. Lai, F. Morle, R. Aebersold, F. J. Dilworth, M. Groudine, and M. Brand. "Activator-Mediated Recruitment of the Mll2 Methyltransferase Complex to the Beta-Globin Locus." *Mol Cell* 27, no. 4 (2007): 573-84.
15. Fossati, A., D. Dolfini, G. Donati, and R. Mantovani. "Nf-Y Recruits Ash2l to Impart H3k4 Trimethylation on Ccaat Promoters." *PLoS ONE* 6, no. 3 (2011): e17220.
16. Tsai, P. H., Y. Chien, M. L. Wang, C. H. Hsu, B. Laurent, S. J. Chou, W. C. Chang, C. S. Chien, H. Y. Li, H. C. Lee, T. I. Huo, J. H. Hung, C. H. Chen, and S. H. Chiou. "Ash2l Interacts with Oct4-Stemness Circuitry to Promote Super-Enhancer-Driven Pluripotency Network." *Nucleic Acids Res* 47, no. 19 (2019): 10115-33.
17. Yang, Z., J. Augustin, J. Hu, and H. Jiang. "Physical Interactions and Functional Coordination between the Core Subunits of Set1/Mll Complexes and the Reprogramming Factors." *PLoS ONE* 10, no. 12 (2015): e0145336.
18. Cho, Y. W., T. Hong, S. Hong, H. Guo, H. Yu, D. Kim, T. Guszczynski, G. R. Dressler, T. D. Copeland, M. Kalkum, and K. Ge. "Ptip Associates with Mll3- and Mll4-Containing Histone H3 Lysine 4 Methyltransferase Complex." *J Biol Chem* 282, no. 28 (2007): 20395-406.
19. Patel, S. R., D. Kim, I. Levitan, and G. R. Dressler. "The Brct-Domain Containing Protein Ptip Links Pax2 to a Histone H3, Lysine 4 Methyltransferase Complex." *Dev Cell* 13, no. 4 (2007): 580-92.
20. Schwab, K. R., S. R. Patel, and G. R. Dressler. "Role of Ptip in Class Switch Recombination and Long-Range Chromatin Interactions at the Immunoglobulin Heavy Chain Locus." *Mol Cell Biol* 31, no. 7 (2011): 1503-11.
21. McManus, S., A. Ebert, G. Salvagiotto, J. Medvedovic, Q. Sun, I. Tamir, M. Jaritz, H. Tagoh, and M. Busslinger. "The Transcription Factor Pax5 Regulates Its Target Genes by Recruiting Chromatin-Modifying Proteins in Committed B Cells." *EMBO J* 30, no. 12 (2011): 2388-404.
22. Sun, J., Y. Zhao, R. McGreal, Y. Cohen-Tayar, S. Rockowitz, C. Wilczek, R. Ashery-Padan, D. Shechter, D. Zheng, and A. Cvekl. "Pax6 Associates with H3k4-Specific Histone Methyltransferases Mll1, Mll2, and Set1a and Regulates H3k4 Methylation at Promoters and Enhancers." *Epigenetics Chromatin* 9, no. 1 (2016): 37.
23. Kawabe, Y., Y. X. Wang, I. W. McKinnell, M. T. Bedford, and M. A. Rudnicki. "Carm1 Regulates Pax7 Transcriptional Activity through Mll1/2 Recruitment During Asymmetric Satellite Stem Cell Divisions." *Cell stem cell* 11, no. 3 (2012): 333-45.

24. Lee, J., D. H. Kim, S. Lee, Q. H. Yang, D. K. Lee, S. K. Lee, R. G. Roeder, and J. W. Lee. "A Tumor Suppressive Coactivator Complex of P53 Containing Asc-2 and Histone H3-Lysine-4 Methyltransferase Mll3 or Its Paralogue Mll4." *Proc Natl Acad Sci U S A* 106, no. 21 (2009): 8513-8.
25. Deng, C., Y. Li, S. Liang, K. Cui, T. Salz, H. Yang, Z. Tang, P. G. Gallagher, Y. Qiu, R. Roeder, K. Zhao, J. Bungert, and S. Huang. "Usf1 and Hset1a Mediated Epigenetic Modifications Regulate Lineage Differentiation and Hoxb4 Transcription." *PLoS genetics* 9, no. 6 (2013): e1003524.
26. Goos, H., M. Kinnunen, K. Salokas, Z. Tan, X. Liu, L. Yadav, Q. Zhang, G. H. Wei, and M. Varjosalo. "Human Transcription Factor Protein Interaction Networks." *Nat Commun* 13, no. 1 (2022): 766.
27. Mayran, A., and J. Drouin. "Pioneer Transcription Factors Shape the Epigenetic Landscape." *J Biol Chem* 293, no. 36 (2018): 13795-804.
28. Balsalobre, A., and J. Drouin. "Pioneer Factors as Master Regulators of the Epigenome and Cell Fate." *Nat Rev Mol Cell Biol* 23, no. 7 (2022): 449-64.
29. Sunkel, B. D., and B. Z. Stanton. "Pioneer Factors in Development and Cancer." *iScience* 24, no. 10 (2021): 103132.
30. Barral, A., and K. S. Zaret. "Pioneer Factors: Roles and Their Regulation in Development." *Trends Genet* 40, no. 2 (2024): 134-48.
